# A Pilot Study on Collective Effects of 22q13.31 Deletions on Gray Matter Concentration in Schizophrenia

**DOI:** 10.1371/journal.pone.0052865

**Published:** 2012-12-28

**Authors:** Jingyu Liu, Alvaro Ulloa, Nora Perrone-Bizzozero, Ronald Yeo, Jiayu Chen, Vince D. Calhoun

**Affiliations:** 1 The Mind Research Network, Albuquerque, New Mexico, United States of America; 2 Department of Electrical and Computer Engineering, University of New Mexico, Albuquerque, New Mexico, United States of America; 3 Department of Neuroscience, University of New Mexico, Albuquerque, New Mexico, United States of America; 4 Department of Psychology, University of New Mexico, Albuquerque, New Mexico, United States of America; University of Illinois at Chicago, United States of America

## Abstract

The association of copy number variation (CNV) with schizophrenia has been reported with evidence of increased frequency of both rare and large CNVs. Yet, little is known about the impact of CNVs in brain structure. In this pilot study, we explored collective effects of all CNVs in each cytogenetic band on the risk of schizophrenia and gray matter variation measured in structural magnetic resonance imaging. With 324 participants’ CNV profiles (151 schizophrenia patients and 173 healthy controls), we first extracted specific CNV features that differ between patients and controls using a two sample t-test, and then tested their associations with gray matter concentration using a linear regression model in a subset of 301 participants. Our data first provided evidence of population structure in CNV features where elevated rare CNV burden in schizophrenia patients was confounded by the levels associated with African American subjects. We considered this ethnic group difference in the following cytoband analyses. Deletions in one cytoband 22q13.31 were observed significantly (p<0.05) more in patients than controls from all samples after controlling ethnicity, and the deletion load was also significantly (p = 1.44×10^−4^) associated with reduced gray matter concentration of a brain network mainly comprised of the cingulate gyrus and insula. Since 80% deletion carriers were patients, patients with deletions also showed reduced gray matter concentration compared with patients without deletions (p = 3.36×10^−4^). Our findings indicate that regional CNVs at 22q13.31, no matter the size, may influence the risk of schizophrenia with a remarkably increased mutation rate and with reduced gray matter concentration in the peri-limbic cortex. This proof-of-concept study suggests that the CNVs occurring at some ‘hotspots’ may in fact cause biological downstream effects and larger studies are important for confirming our initial results.

## Introduction

Schizophrenia (SZ) is a severe mental disorder marked by hallucinations, delusions and cognitive deficits with a heritability estimated at 73–90% [1]. Genetic factor plays a major role in liability for this disorder. While many genes and SNPs have been identified to be associated to SZ, copy number variation (CNV) recently became a new focus of genetic studies. As a specific type of DNA structural variation, CNVs alter more nucleotides than any other type of genetic variations [2], constitute up to 5% of the whole genome [3], and involve multiple genes and/or regulatory regions. Not surprisingly new findings have emerged regarding the effect of CNVs on the risk for SZ [4]–[9].

Several chromosomal regions with copy number mutations have been repeatedly associated with schizophrenia. For example, 22q11.2 deletion syndrome (also known as DeGeorge syndrome) caused by a deletion of about 3 million base-pairs (bp) affects almost every system in the body and is associated with heart problems [10], mild facial dysmorphology [11] and the risk for SZ [12], [13]. The association of this deletion along with microdeletions at 1q21.1 and 15q13.3 with SZ has been replicated in separate studies [14]–[16]. Additionally, large CNVs (>100 k bp) at specific regions involving genes in important neuro-developmental pathways, such as *NXRN1, ERBB4* and *CNTAP2* [15], [17]–[19], have hinted to the involvement of developmental alterations in this illness. In addition to these individual large CNVs, the total CNV burden across the whole genome has also been associated to many phenotypic variations [20]–[23], including schizophrenia with elevated frequencies of large CNVs, rare CNVs, and rare deletions in patients [19], [23].

Although much evidence attests to the impact of CNV’s on schizophrenia, the neurobiological effect of CNVs remains poorly understood and studies have yet to identify the specific neurobiological mechanisms by which these genetic variations influence brain anatomy or development in schizophrenia. An initial way to address this issue would be to investigate the effect of CNVs on structural or functional brain phenotypes associated with schizophrenia. In the current study we analyzed gray matter variation measured by structural magnetic resonance imaging (MRI). Structural MRI studies have provided some of the most consistent evidence for brain abnormalities in schizophrenia. SZ patients have shown specific deficits in gray matter volumes, especially in frontal, temporal regions [24], as well as striatal and limbic regions as reviewed in several publications [25]–[27]. There is also sufficient evidence to show the use of structural MRI as a phenotype to investigate genetic effect on schizophrenia [28].

Large individual CNVs as mentioned above hold great interests for SZ studies, yet they are quite rare, occurring in less than 1% of SZ patients [12], [29], and hence, their overall impact on the risk for schizophrenia is likely quite modest. Here, we want to investigate all possible SZ related CNVs, including the collective effects of small CNVs with size about 1 kbp [30] on gray matter variation. Because the total CNV burden across genome provides a rather gross measure of CNVs, we explored the collective effect of all CNVs within each of 811 cytogenetic bands according to NCBI36/hg18 genome map. Cytogenetic bands are not arranged randomly in chromosomes, instead nucleotides within each cytoband share similar GC contents and genes within each cytoband share similar expression breadth [31]. We hypothesized that CNVs occurring across some specific chromosomal regions such as cytogenetic bands at a different rate in SZ patients, could collectively be associated with brain structural phenotypes, in particular gray matter reduction in SZ patients.

## Results

In this study, we analyzed CNV profiles from 324 participants (151 patients with schizophrenia and 173 healthy controls) and gray mater concentration (GMC) images from a subset of 301 participants (140 patients and 161 controls). For replication purpose, we first presented the CNV profiles, including large CNVs, CNVs in three hot spots associated with SZ, and total CNV burden. Then we identified the CNV features in each cytobands that showed difference in SZ patients. Finally, we reported the association results of such CNV features with GMC loadings in different brain networks.

### 2.1 CNV Profiles and Distribution Across Subjects

We analyzed the overall CNV incidence distribution among 324 participants, as listed in Table 1. The length of CNVs ranged from 418 bp to 778 K bp, and only 6 CNVs (1q31.2, 2q32.1, 9p21.3, 11p14.3, 11q22.1, 16p11.2) were larger than 500 k bp. Five of the six large CNVs were rare incidence and four of the six were (3 deletions at 9p21.3, 11q22.1, 16p11.2 and an insertion at 1q31.2) from the SZ patient group. The deletion at 11q22.1 affected the *CNTN5* gene and the deletion at 16p11.2 has been reported to be associated with SZ and autism [14], [32], [33]. Additionally, we checked three hot spots previously reported to be associated with SZ: 1q21.1, 15q13.3 and 22q11.21. As shown in Table 2, two deletions at 1q21.1 were observed in two SZ patients. They affected the exact same region (143.38 M–144.47 M bp) as reported in [15] and one had a relatively large size of 368 k bp. At 15q13.3, four small deletions (30,297,184–30,303,141 bp) occurred in one patient and three controls, but they did not interrupt the *CHRNA7* gene. At 22q11.2, 13 CNVs were observed, but most were small mutations and none affected key genes previously implicated in SZ (*COMT, PRODH,GNB1L, DGCR2,PIK4CA* and *DGCR8* [12]).

**Table 1:** Overall CNV distribution in 324 samples.

	Total CNV statistics	Rare CNVs (<1%)	Frequent CNVs (>1%)
Deletions/Insertions	33692/31023	1564/231	32128/30792
Length of CNVs	418 bp to 778,212 bp; 6 CNVs >500 kbp	418 bp to 778,212 bp; 5 CNVs (3 deletionsand 2 insertions) >500 kbp	501 bp to 516,868 bp; 1 CNV(deletion) >500 kbp
Median length of Deletions/Insertions	1884 bp/1989 bp	2838 bp/5983 bp	1870 bp/1954 bp

**Table 2:** Specific CNV regions distribution.

Previous reported CNVs regions in [15]	SZ (151 subjects)		HC (173subjects)	
	Deletion:#(length)	Insertion:#(length)	Deletion:#(length)	Insertion:#(length)
*1q21.1(142.5–145.5* *M*)	1(6.5 k bp), 1(368 k bp)	None	None	None
*15q13.3 (28–31* *M)*	1(6.0 k bp)	None	3(6.0 k bp)	None
*22q11.21 (17–20.5* *M) main 22q11DS region*	2(2.3 k bp)	2(1.2 k bp)	1(28 k bp), 2(2.3 k bp)	1(113 k bp), 1(5.3 k bp), 3(1.2 k bp), 1(959 bp)

To determine whether overall CNV burden differed between patients and healthy controls, we compared metrics of total CNV incidence, total deletion, total rare CNVs and total rare deletion using two-sample t-tests. Since relatively more African Americans (AA) were recruited into the patient group than the control group, we compared the AA vs. other ethnic groups, and re-tested schizophrenia vs. control differences with ethnicity as a covariate. Results (in Table 3) indicate significantly more CNV incidences were observed in the AA group. Although more rare CNVs and rare deletions were found in patients than controls with p values less than 0.05, this higher rate was confounded by ethnicity. When ethnicity was modeled as a covariate in an n-way ANOVA test together with diagnosis, the patient group did not show significantly different CNV burden from controls. Because DNA was extracted from blood or saliva, we tested the potential influence of tissue type and found that whether or not modeling the tissue type as a covariate did not change results.

**Table 3:** Total CNV burden effects in groups.

Total CNV burden effect	SZ vs. HC (t-test on all samples)		AA vs. others		SZ vs. HC (ANOVA with ethnicity, tissue type and diagnosis as covariates)	
	P value	T	P value	T	P value	F
Total CNVs	0.47	−0.73	0.53	0.63	0.35	0.87
Total deletions	0.26	−1.13	0.0008	−3.4	0.47	0.51
Total rare CNVs	0.05	**1.96**	8.21×10^−60^	20.36	0.73	0.12
Total rare deletions	0.02	**2.44**	7.31×10^−66^	21.93	0.30	1.05

### 2.2 Cytoband CNVs Distribution Across Subjects

We summed individual participant’s CNVs within each of the 811 cytogenetic bands, specifically counting the number of CNVs, the number of deletions and the number of insertions within each cytoband. 641 cytobands showed at least one CNV mutation among 324 subjects. Two-sample t-tests on these CNV measures identified no single cytogenetic band responsible for group differences that can pass a Bonferroni multiple comparison correction, which requires a p value less than 7.8×10^−5^ (0.05/641). Given the sparse nature of CNV incidences, it was expected that some of cytogenetic bands with uncorrected p<0.01 may still hold potential biological functional impact. There were 17 cytobands with such different CNV ratios in SZ patients from controls, and among them 14 cytobands presented the same SZ difference when considering only the White samples (270 White samples, uncorrected p<0.05). Thus, these 14 cytobands listed in Table 4 were selected for further association analyses with GMC of brain networks.

**Table 4:** Number of cytobands showing potential difference in patients (uncorrected p<0.01).

Cytoband CNVs*	CNV incidences	Deletions	Insertions
Number of bands with p<0.01 (uncorrected)	6q12; 7p12.3; 8q11.22; 8q24.23; 9p13.2;16p13.3; 22q13.31	1p22.1; 2p24.3; 7p12.3; 7q32.1;22q13.31	2q12.3; 2q23.3; 5q21.1; 8q11.22; 15q22.2;

* : these 17 CNV features come from 14 cytogenetic bands, while 3 bands show difference in both CNV incidences and deletions or insertions.

### 2.3 Effects of Cytoband CNVs on Brain GMC

From the 301 participants’ structural GMC images, we extracted 18 spatially independent brain networks by independent component analysis (ICA). A brain network is a data driven ROI with regions co-varied across samples clustered together. These 18 brain networks comprised 98.34% of total structural GMC variance, and included all main brain regions (see Figures S1, S2 for the plots of 18 networks). Among the 18 brain networks, three networks (superior temporal and inferior/medial frontal network, superior/middle frontal network, and precuneus/cuneus occipital network) showed significant differences between SZ patients and controls after controlling age, gender and scan sites, with p values passing Bonferroni correction (<0.002 see Figure S1). We also tested possible medication effects on these brain networks in patients using a linear regression model with chlorpromazine equivalent medication dosage [34], age, gender and sites as independent variables, and found no significant effects of medication in any of these brain networks.

**Figure 1:**
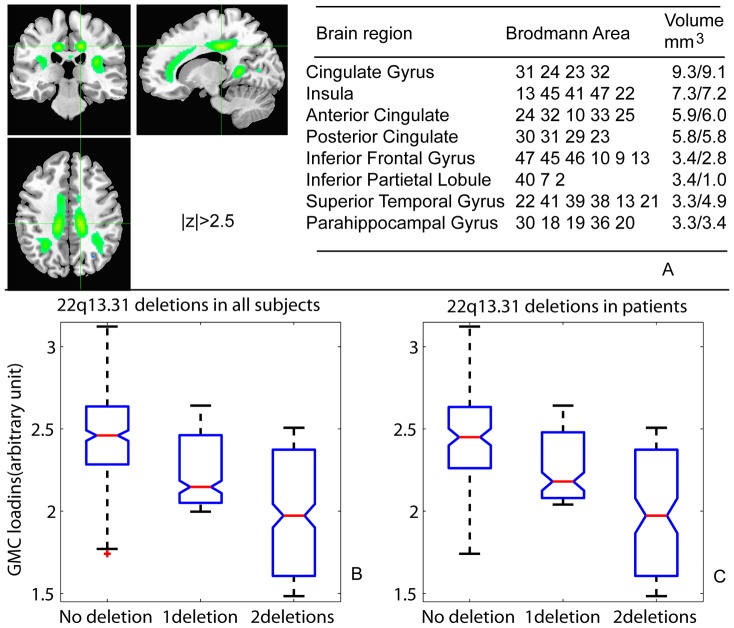
22q13.31 association with brain GMC. a) The brain network mainly locates in cingulate gyrus, insula and parahippocampal gyrus. We highlighted the regions with normalized weight z >2.5 in green color. b) Subjects carrying different deletion numbers in 22q13.31 show different level of loadings in this GMC network. The more deletions a subject carries, the lower the subject’s loading on this GMC network is. C) In SZ patients, the deletion loads also significantly correlated to GMC loadings in this brain network. Patients with deletions show significantly reduced GMC in this brain network compared to patients without deletions.

**Figure 2:**
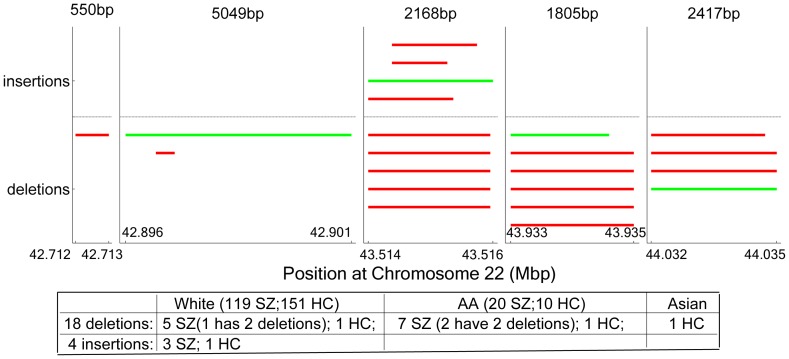
22q13.31 CNVs distribution. Each solid line presents one CNV incident with corresponding starting position and ending position. Red lines indicate CNVs from SZ samples, and green lines indicate CNVs from HC samples. 18 deletions are drawn below the neutral dash line and 4 insertions are drawn above the neutral line. The length of CNVs is not scaled equally, but corresponds to the text above the plots.

We then hypothesized that CNV features distinguishing groups (14 cytobands differing in SZ patients with p<0.01) would affect brain structure. A general regression model was used to test these CNVs’ effect, where the loadings of each brain GMC network are the dependent variable, and independent variables include age, gender, scan site, and a CNV feature. Among the 14 cytobands, only deletions at 22q13.31 showed a significant correlation with the GMC loadings of a brain network. This brain network mainly comprised of the cingulate gyrus (anterior, mid- and posterior cingulate gyri), insula, inferior frontal gyrus and parahippocampal gyrus, as highlighted with green in Figure 1a. In the regression model, deletions at 22q13.31 explained 5% of total variance of GMC in this brain network with a p-value of 1.44×10^−4^ (passing Bonferroni correction of 0.05/18/14). The second smallest p value of connections between tested cytobands and all brain networks was 0.003, followed by 0.02. Therefore, the association between 22q13.31 deletions and the GMC in the cingulate-insula network is remarkably strong. To further illustrate the effect of these deletions on this brain network, we plotted the cingulate-insula network’s loadings in 301 subjects against loads of deletions at 22q13.31 in Figure 1b. Deletion carriers in general have lower loadings of GMC in this brain network than the no deletion carriers (ANOVA test p value of 3.36×10^−4^ for three deletion groups). The one-deletion group and the two-deletion group showed, on average, 9% and 19% reductions of GMC loadings, respectively, compared to the no deletion group. Since 80% of these deletion carriers were SZ patients (see Figure 2), we also tested the GMC loading difference between patients with deletions and patients without deletions. A very similar result was derived in Figure 1c, where SZ patients with deletions showed significant lower GMC loading compared with SZ patients with no deletions (p = 6.10×10^−3^, the patients with one deletion and those with two deletions showed, on average, 8% and 19% reductions of GMC loadings, respectively, compared to the no-deletion patients). The three healthy controls carrying one deletion also showed a similar reduction of gray matter loadings (10% on average). For completeness, we also examined the effect of the 4 insertions observed at 22q13.31, and found no significant differences in the GMC loadings of those cases (p = 0.60).

### 2.4 Analysis of CNVs at 22q13.31

At the 22q13.31 band, we observed 22 CNVs (Figure 2, Table S1) among 324 participants, including eighteen deletions occurring in twelve SZ patients (three patients with two deletions apiece) and three healthy controls, and four insertions occurring in three patients and one healthy control (see Figure 2). The ethnic attribute of these 22 CNVs was also reported in Figure 2. Using ethnicity as an additional covariate in an ANOVA test, the CNVs and deletions were both significantly more frequent in patients than healthy controls with p<0.05. Different tissue type did not affect this cytoband CNVs (p>0.77). All the 22q13.31 deletion carriers did not harbor any relatively large CNVs (>100 kbp) in the genome, thus the effects of this variation on SZ and brain structure was less likely influenced by other CNVs.

The size of the 22 CNVs ranged from 418 bp to 5000 bp, with two CNVs smaller than 1 kbp. From the starting position of the first CNV to the ending position of the last CNV, 1.32 M bps were included (chromosome position 42,712,496 to 44,035,019). This region partially overlaps with the terminal deletion of 22q13.3, which covers several Mbp counting back from the end of chromosome 22. The specific genes affected by the deletions at this region in our sample include *SAMM50*, *PARVB*, *PARVG*, *ARHGAP8* and *LOC100506714*, and does not affect the key gene *SHANK3* [35] in the terminal deletion of 22q13.3.

### 2.5 Verification CNV Calls by Real-time PCR

As shown in Figure 2, five regions at 22q13.31 contain CNVs. The CNVs in the first 4st four regions detected by genome wide genotyping data were further validated using TaqMan® real-time PCR with primers specific to each region. Real-time PCR results confirmed the presence of deletions in 13 out of 14 deletion calls detected by genomic genotyping data. One not confirmed deletion presented a low confident call in the PCR result. Four insertions in the 3^rd^ region were not confirmed by PCR, implying the difficulty in detecting insertions using genotyping intensity values (Log R ratio) [3], [40], [41]. Within the verified deletions, we still observed the SZ related difference (p = 0.0056) and the connection with the cingulate-insula network GMC (p = 1.58×10^−4^).

## Discussion

Within the 324 participants’ genomic CNV profiles derived from Illumina 1 million assays, we observed copy number variations that are, in general, smaller than CNVs reported in [19] and larger than CNVs reported by Glessner, et al. [30]. This difference reflects the use of different resolution arrays [36]. In our sample, rare CNV and rare deletion burden were elevated in individuals with schizophrenia, but this higher rate was confounded by ethnicity, with the AA subjects showing significantly higher rates of rare CNVs and rare deletions than other subjects. After controlling for ethnicity, we did not observe any significant difference of the total CNV burden in SZ patients vs. controls, which could be due to our small sample size.

For replication of previously reported CNVs in literature, we particularly studied large CNVs (>500 kbp) and CNVs in three regions, 1q21.1, 15q13.3 and 22q11.21. In our data large CNVs were more frequent in patients than controls (4 out 6 large CNVs were in patients), supporting the hypothesis of the involvement of large, rare CNVs in the pathogenesis of schizophrenia [37]. Specifically, important may be the deletion at 11q22.1 we observed. This deletion affects the *CNTN5* gene **(**encoding contactin 5 protein) that plays a role in the formation of axon connections in the developing nervous system and has been suggested to be involved in autism and schizophrenia [38]. Another deletion we observed at 16p11.2 involves many genes and this CNV region’s association with schizophrenia and autism has been replicated in several studies [14], [32], [33]. From the three hot regions, we observed one relatively large rare deletion (368 k bp) at 1q21.1 in one SZ patient. Smaller CNVs occurred at these regions, particularly at 22q11.2, yet they do not interrupt key genes (*CHRNA7, COMT, PRODH, GNB1L, DGCR2, PIK4CA* and *DGCR8*) reported by Bassett et al. [12]. Again, our data support the involvement of large rare deletions, although we did not observe any large deletions at 15q13.3 and 22q11.21, perhaps because of our relatively small sample size. Because of such large CNVs’ rareness (only one sample per CNV in our data) we did not test their impact on brain structure in our data.

To investigate possible impacts of CNVs on brain structural variation, we evaluated 14 cytoband CNVs that showed potential relations with SZ, in conjunction with 18 GMC brain networks. Only deletions at one cytoband, 22q13.31, exceeded the stringent significance level for an effect on the GMC in the cingulate-insula network (Figure 1a). Similar brain regions can be derived from GMC images using a voxel-wise regression model with the deletions as one regressor (see Figure S3), but only sparse voxels can pass multiple comparison corrections. In contrast, the brain network derived from ICA provides clear continuous regions with less sparse voxels. In Figure 1b-1c, the deletion load at 22q13.31 was significantly, negatively correlated to GMC loadings of this brain network in all samples and SZ patients only. Since 80% of deletions occur in patients, the difference between patients with deletions and without deletions provides more insight into the CNVs’ impact on the brain structural abnormality of patients. Figure 1c clearly demonstrates that patients with deletions have lower GMC loadings in this particular brain network, reflecting a lower gray matter concentration in cingulate gyrus and insula, compared with patients without deletions.

A recent meta-analysis documents gray matter reductions in the cingulate gyrus and insula in patients with schizophrenia [26]. In our data, although 22q13.31 deletion carriers clearly showed reduced gray matter concentration in the cingulate-insula network, and 80% of deletion carriers were SZ patients, we did not observe a significant difference in this brain network between the patient group and healthy controls. We believe that this may indicate genetic heterogeneity within the SZ patient group. Schizophrenia is known to be complex and heterogeneous from both genetic and clinical viewpoints. Genetic vulnerability interacting with environmental stress leads to a clinical syndrome comprised of positive, negative and cognitive symptoms. Our data and ANOVA tests suggest that two different patient groups, with and without deletions at 22q13.31, showed significant different pattern of gray matter concentration in the cingulate–insula network. This genetic heterogeneity may limit the specific gray matter reduction effect on the SZ patient group. As illustrated in our sample, patients with deletions clearly showed reduced gray matter concentration in this brain network, but this was not evident in the entire patient group. This finding suggested that genetic trait in conjunction with brain structural variation could provide an important tool to subcategorize SZ patients.

Across 22q13.31 cytoband, more CNVs, particularly deletions, were observed in patients than healthy controls with a significant level (p<0.05) after controlling ethnicity. These are small copy number mutations with size from about 500 bp to 5 k bp. Compared with the terminal 22q13.3 deletion, which extends from approximately 41.12 M ∼ 49.47 M bp to the end of chromosome 22 at 49.57 M, our CNVs deletions partially overlap with the beginning part of the typical 22q13.3 deletion, but do not affect the key *SHANK3* gene [35] located at the end of the chromosome (49.46 M - 49.52 M bp). Therefore we do not believe the small deletions at 22q13.31 seen in our sample are part of the 22q13.3 deletion. Rather our results suggest that 22q13.31 is a very active region for breaking points of CNVs, including both small deletions and large 22q13.3 deletions. These small deletions at 22q13.31, collectively, cover 1.32 M bp and affect multiple genes and intergenic regions. These affected genes include *SAMM50, PARVB, PARVG*, and *ARHGAP8*, involved in basic cell functions such as cell energy, cell growth and death, cell adhesion, and cell migration. Although not all the deletions affect the same genes, this whole region can be critical for normal brain development, and disruption of it could affect pathogenesis of neurological disorders. To the best of our knowledge, little is known about how these genes in this region are associated with SZ. However, the current data reveal that the presence of these deletions is significantly associated with SZ and GMC reduction primarily in the cingulate gyrus and insula, explaining 5% of GMC variation in this network. ANOVA test clearly demonstrated that patients with deletions show lower loadings of GMC in this network than patients without deletions.

One main limitation of this study is the relatively small sample size for CNV analyses, which may explain why the increased number of total rare CNVs observed in patients vs. controls did not reach statistical significance. Despite the small sample size, we believe that the cytoband analyses used here facilitate statistical power for two specific reasons: 1) the penetrance of genetic variations is high in neuroimaging phenotypes compared to diagnoses [39], and 2) we studied the collective effects of CNVs from 641 cytogenetic bands, not the much more numerous and sparse individual CNVs across genome. Nevertheless, this is a pilot study on cytoband CNVs’ impact on brain structure, and larger studies will be important for confirming these initial results. Other concerns from the use of both blood and saliva samples and images scanned from different sites were also considered in this study. Patients and controls were included at all sites and from both tissue types to allow corrections for the potential effect. A correction method for LRR data to eliminate DNA quality difference was applied. Verification of tissue type effect on CNV features and regression of site effects from imaging data were implemented to address these issues. We believe that this approach is able to remove a significant portion of possible impact on the findings. In addition, our real-time PCR verification showed that deletion calls by inferring genotyping arrays are very reliable, with 13 out of 14 verified by PCR. Insertions were not verified indicating the difficulty in calling insertions by genotyping LRR data, which has been observed in [3], [40], [41]. Because our findings focused on deletions, the results still hold for the verified CNVs.

This study is a proof of concept for the collective effect of CNVs at specific regions on altering gray matter in selected regions of peri-limbic cortex in schizophrenia. Our data suggest that, collectively, even small CNVs may influence the risk of schizophrenia, with a remarkably increased rate of copy number mutations at 22q13.31, and with reduced gray matter concentration in brain regions, such as the cingulate-insula network critical for emotion processing and a central hub in the brain’s default mode network [42]. Our findings also indicate the heterogeneity of SZ patients from a genetic viewpoint; as patients with deletions show significant reduction in gray matter concentration in the cingulate-insula brain network than patients without deletions. Given our relatively small sample size and the even smaller number of deletion carriers, our findings, undoubtedly, need further replication in larger sample sizes. However, we believe, our findings provide evidence that CNVs no matter the size, occurring at some ‘hot’ regions, may in fact cause biological downstream effects, and are worthy of in-depth investigation.

## Materials and Methods

### 4.1 Participants

This study combined data from two center studies: the Mind Clinical Imaging Consortium (MCIC), a multisite collaborative study (University of New Mexico-Mind Research Network, Massachusetts General Hospital, University of Minnesota, University of Iowa) of first-episode and chronic schizophrenia patients; and the Center for Biomedical Research Excellence (COBRE), a multidisciplinary study on brain function and mental illness hosted at the University of New Mexico-Mind Research Network. The institutional review boards at each site (Universities of Iowa, Minnesota, and New Mexico and Massachusetts General Hospital) have approved the studies and all participants provided written informed consents after complete description of the study to the participants. All potential participants who declined to participate or otherwise did not participate were not disadvantaged in any other way by not participating in the study. The MCIC patient group comprised subjects that met DSM-IV-TR criteria for schizophrenia, schizophreniform disorder, or schizoaffective disorder. The diagnoses were based on DSM-IV criteria using the Structural Clinical Interview for DSM Disorders (SCID). Patients were excluded if they had a history of neurologic or psychiatric disease other than schizophrenia, head injuries, lifetime history of substance dependence or abuse within the past month, or an IQ less than or equal to 70. The MCIC controls were screened using the SCID, and subjects were excluded who were diagnosed with substance abuse/dependence, medical, psychiatric, or neurological illnesses. Healthy controls were not excluded if they had been medicated with antidepressants, antianxiety, or sleep deprivation medications, so long as these medications had not been taken for at least 6 months prior to the scan and for not more than 2 months of continuous use at any time [43]. Similarly, the COBRE patient group comprised schizophrenia patients screened using DSM-IV criteria. The healthy control group included participants with no history of neurological or psychological disorder screened by SCID.

A total of 334 subjects were recruited at the time of this project, 324 subjects had good quality genotyping data for CNV analyses and 301 subjects also provided good quality structural MRI images. The demography information of these subjects is listed in Table 5. The 324 subjects with CNV data came from different ethnic groups, including White, African American, Asian, Native American and Pacific Islander. There were 151 SZ patients and 173 healthy controls. We noticed that in the AA group significantly more SZ patients were recruited compared to controls. This ethnic bias may confound the patients’ group difference in the CNV data, if the CNV data have population differences regarding African American. We considered this potential confounding effect when analyzing and interpreting results. In the 301 subjects providing also structural MRI images, there is no difference in age and sex between patients and controls.

**Table 5:** Demographic information of 324 CNV participants and 292 MRI participants.

324 CNV subjects	Male		Female		White	African American	Asian		Pacific Islander	Native American	Unreported
SZ	115		36		119	20	6		1	0	5
HC	112		61		151	10	5		1	1	5
**MRI subjects**											
**301 MRI subjects**		**Male**	**Female**	**Age**	**White**	**African American**	**Others**	**Collecting Sites**			
								**NM**	**Minnesota**	**Harvard**	**Iowa**
SZ		106	34	36±12	110	19	11	51	30	28	31
HC		104	57	33±11	142	8	11	59	19	23	60

### 4.2 Genotyping and CNV Calls

250 subjects from MCIC study provided whole blood samples and 84 subjects from COBRE (six subjects participated in both studies) study provided saliva samples. The genetic lab at Mind Research Network conducted DNA extraction and genotyping for the blood or saliva samples using the Illumina Infinium HumanOmni1-Quad assay, following the industry recommendations. No significant difference exists between genotyping call rate of saliva and blood samples, and the large variation observed in the intensity value (Log R Ratio: LRR) from salvia sample was corrected in the data correction step. Briefly, LRR data correction was performed including correction for extreme outliers, principal components associated with GC (guanine-cytosine)-content [44], DNA quantity and ethnicity. After the correction, no difference exists in the quality of LRR data from saliva or blood DNAs measured by LRR standard deviation [45]. Then, quality control based on LRR standard deviation (δ<0.28 [45]) was applied. The qualified LRR, β allele frequency (BAF), and genotype from about 1 million SNP/CNV loci were segmented using a circular binary segmentation algorithm [46] and a hidden Markov model algorithm (PennCNV [47]) independently. Only segments (spanning at least 3 markers) detected by both algorithms (segments overlapping or apart by less than 3 markers) went through a single to noise ratio check calculated by the ratio of the segment mean LRR over neighboring LRRs to make the final CNV calls. CNV calls overlapping with telomere or centromere larger than 50% were excluded, as well as small segments with less than 500 bp (one exception is a homozygous deletion at 22q13.31 with 418 bp). The detail of this conservative CNV calling pipeline can be found in Text S1 and [48], [49]. Four samples with total CNV calls exceeding three standard deviations were excluded. If two CNV calls from different samples overlapped or the distance between CNVs was less than three markers, we treated them as from a common CNV region. The region with less than 1% CNV frequency among subjects was defined as a rare CNV region. For each CNV region, subjects can have deletion (copy number 0 or 1), neutral (copy number 2) or insertion (copy number 3 or more).

### 4.3 Structural MRI

The structural images (*T*_1_-weighted MRIs) were collected at each site using 1.5T scanners at Harvard (Siemens), New Mexico (Siemens) and Iowa (GE) and a 3T scanner at Minnesota (Siemens). Imaging parameters for the scans at Harvard and New Mexico were TR/TE = 12/4.76 ms, slice thickness = 1.5 mm, bandwidth = 110 Hz, voxel dimensions = 0.625×0.625×1.5 mm. At Iowa the parameters were TR/TE = 20/6 ms, slice thickness = 1.6 mm, bandwidth = 122 Hz, and voxel dimensions = 0.664×0.664×1.6 mm. At Minnesota the parameters were TR/TE = 2530/3.81 ms, slice thickness = 1.5 mm, Bandwidth = 110 Hz, voxel size = 0.625×0.625×1.5 mm [43]. All scans were collected in a coronal orientation. The scan site effect on MRI images has been observed [43] and we considered this effect in our regression model by adding the sites as additional independent dummy variables. The MRI images were preprocessed using the voxel based morphometry (VBM: [50]) in Statistical Parametric Mapping 5 (SPM5) software (http://www.fil.ion.ucl.ac.uk/spm/software/spm5/). We applied optimized VBM where tissue classification, bias correction, and image registration are integrated within a unified model. Unmodulated normalized parameters were used for segmentation to segment the brain into white matter, GM, and cerebral spinal fluid probabilistic maps. This unmodulated gray matter image presents gray matter concentration for each voxel [51] in the brain, termed a GMC image. The voxel size for all images was resliced to 2×2×2 mm as SPM template. A quality check to remove images four standard deviation away from averaged GMC image of each group was applied (two GMC images were removed). Finally, 301 GMC images were further analyzed in this study.

### 4.4 Association Analyses

Various types of CNV metrics were calculated, including the total number of CNVs, deletions, rare CNVs, and rare deletions from the whole genome, and the number of CNVs, deletions and insertions from each cytogenetic band. We tested their potential SZ relatedness using a two-sample t-test between SZ patients vs. healthy controls. Since there was a potential ethnicity confounding effect, we further tested whether African American population showed difference using a two-sample t-test between AA group vs. all others, and re-tested SZ difference with ethnicity and tissue type as covariates. For the 811 cytogenetic bands across the whole genome, the number of CNVs occurring within each cytoband in our sample was wide spread from 0 to 324, with median of 17 skewed to the lower end. Only 641 cytobands had at least one CNV incidence. Such sparse nature of CNVs diminishes the statistical power, violates the normal distribution assumption in t-tests and makes the multiple comparison correction for 641 cytobands too stringent. We, therefore, did not apply Bonferroni correction, instead assumed that any cytoband showing different CNV ratio in SZ patients tested by two sample t-tests with uncorrected p<0.01 and the same SZ difference in the White sample only may have the potential to affect SZ, and thus was selected for association analyses with brain GMC features. Possible ethnicity and tissue type effects on the CNVs at selected cytobands were also tested using an n-way ANOVA model including ethnicity and tissue type as covariates.

Specific GMC features were extracted by ICA from GMC images, where ICA is a well established method for identifying independent brain networks [52], [53]. ICA can be expressed in a general multivariate linear model, X = AS, where X is the observation matrix, S is the independent component matrix and A is the loading matrix. The algorithm optimizes the A (or its inverse W) matrix to extract the maximally independent latent components embedded in the observations. In our application, X is GMC images from all subjects forming a subject-by-voxel matrix; S is the spatially independent brain networks embedded in all subjects images. Each independent brain network is a GMC feature comprised of several brain regions co-varied together across subjects and maximally independent from other networks [24]. The A matrix represents how each brain network expressed in subjects. In this study, we implemented the GIFT (http://mialab.mrn.org/software/) build-in infomax ICA method [54]. The number of components embedded in the GM structure was estimated through a minimum description length method [55] on uncorrelated voxels. The component number from 15 to 22 all produced similar and significant results and we chose the middle number 18 in this report, which explained more than 98% of total variance in the GM structure. Therefore, 18 independent components (brain networks) were analyzed for association with cytoband CNVs. Each brain network has its corresponding loadings on subjects, where a high loading on a subject means that the subject has a high level of GMC in the brain network. The scanner difference was specifically considered in the following regression model.

The association between selected cytoband CNV metrics and 18 GMC brain networks was assessed by a linear regression model. In this model, the dependent variable is the loadings of a GMC component on subjects and the independent variables include age, gender, scan sites (3 dummy variables), and a CNV metric. Bonferroni correction for 18 brain networks and selected CNV metrics was applied to the significance of regression coefficient from the CNV metric to the GMC loadings.

### 4.5 TaqMan® Real-time PCR Verification

Due to limited DNA quantity for each sample, we cannot verify all CNVs using real time PCR. We selected four regions from cytoband 22q13.31 containing CNVs detected by genotyping arrays. For each CNV region we designed a specific TaqMan primer set. Following TaqMan® protocol, a TaqMan® copy number reference assay RNase P was run simultaneously with specific primer sets as the reference of being two copies. CopyCaller software was used to make copy number calls.

## Supporting Information

Figure S1 **The 1^st^ to 12^th^ brain networks extracted from GMC images, thresholded at Z>2.5.** The 9^th^, 10^th^ and 12^th^ brain networks show significant differences between schizophrenia patients and healthy controls after controlling age, gender and sites with p values of 1.53E−14, 1.76E−4 and 3.58E−7, respectively. The 9^th^ network mainly includes medial and inferior frontal gyri, superior temporal gyrus and anterior cingulate. 10% of the variation in this network is explained by the patient and control group difference. The 10^th^ network is mainly in precuneus, cuneus, and occipital gyri, and the 12^th^ network is in superior and middle frontal gyri.
(TIF)

Figure S2 **The 13^th^ to 18^th^ brain networks extracted from GMC images, with a threshold of Z>2.5.**
(TIF)

Figure S3 **the GMC map associated to deletions at 22q13.31 using a voxel-wise linear regression model.**
(TIF)

Table S1 **22q13.31 CNVs’ chromosome position (hg18/NCBI36).**
(DOCX)

Text S1 **the CNV detection procedure.**
(DOCX)
