# Supplementary figures and images for: A Pilot Study on Collective Effects of 22q13.31 Deletions on Gray Matter Concentration in Schizophrenia
Source: PLoS One. 2012 Dec 28;7(12):e52865. doi: 10.1371/journal.pone.0052865 (PMC3532105; doi:10.1371/journal.pone.0052865)

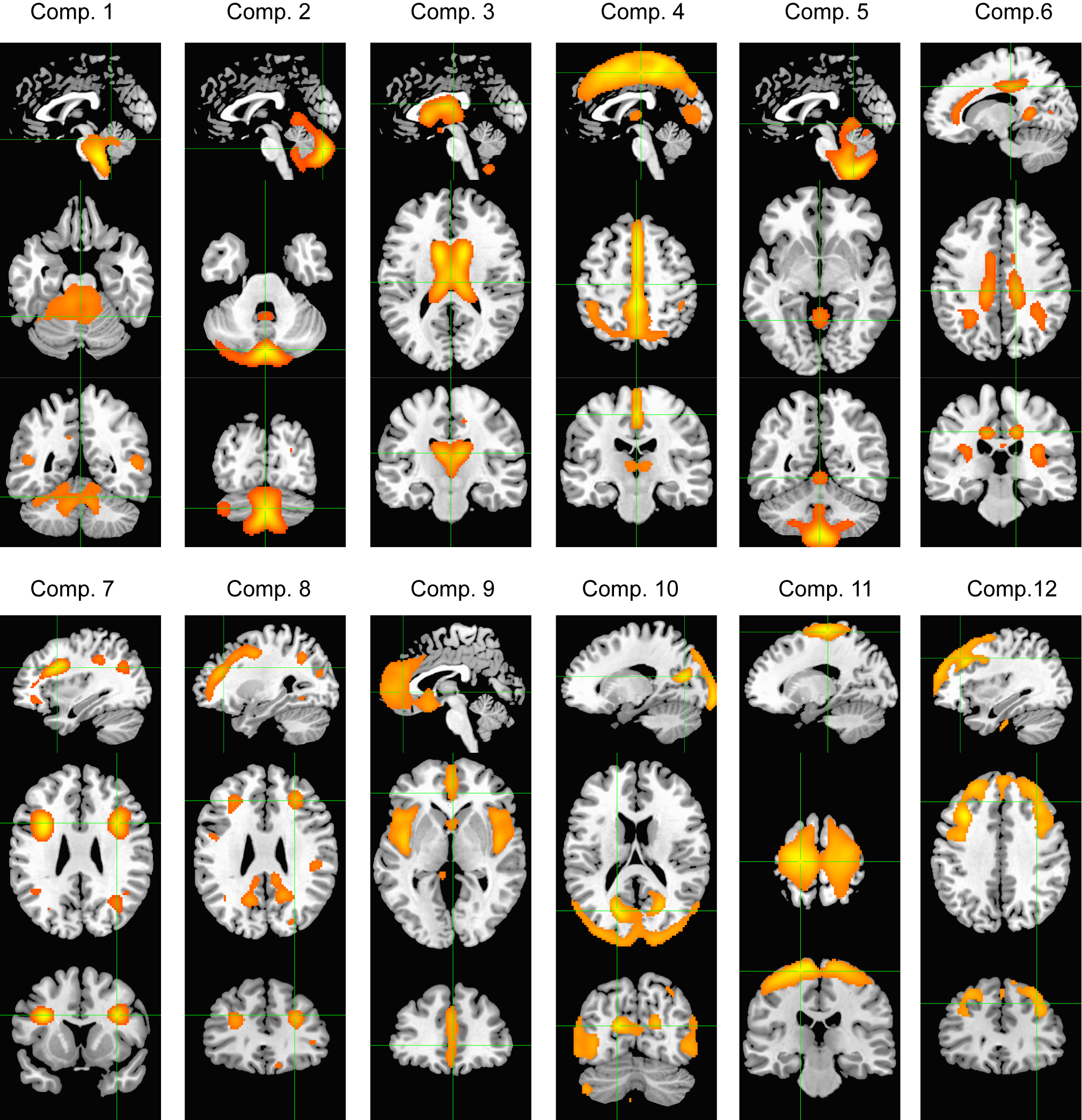

Supplement: Figure S1 — The 1st to 12th brain networks extracted from GMC images, thresholded at Z>2.5. The 9th, 10th and 12th brain networks show significant differences between schizophrenia patients and healthy controls after controlling age, gender and sites with p values of 1.53E−14, 1.76E−4 and 3.58E−7, respectively. The 9th network mainly includes medial and inferior frontal gyri, superior temporal gyrus and anterior cingulate. 10% of the variation in this network is explained by the patient and control group difference. The 10th network is mainly in precuneus, cuneus, and occipital gyri, and the 12th network is in superior and middle frontal gyri. (TIF) [file pone.0052865.s001.tif]

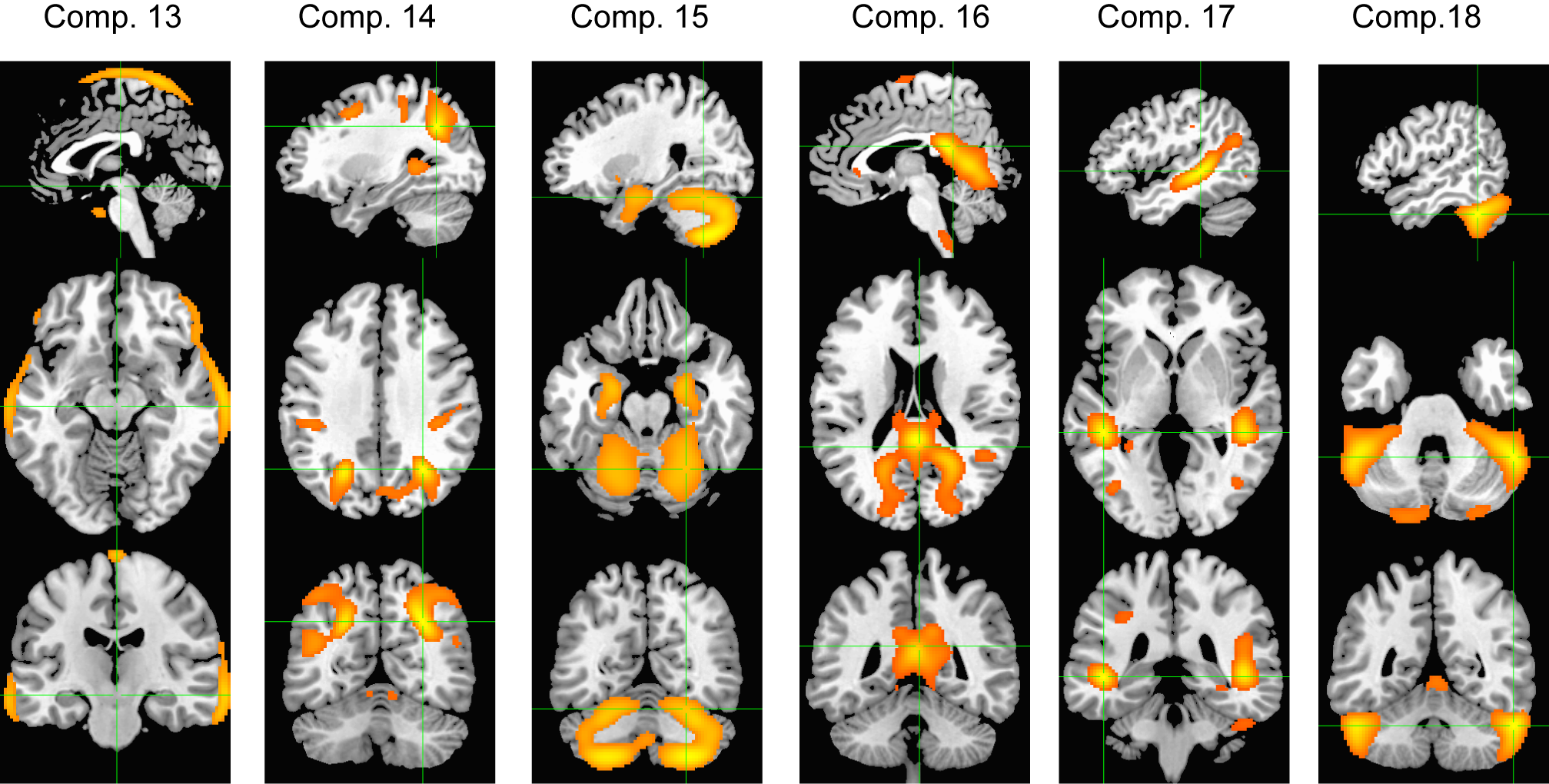

Supplement: Figure S2 — The 13th to 18th brain networks extracted from GMC images, with a threshold of Z>2.5. (TIF) [file pone.0052865.s002.tif]

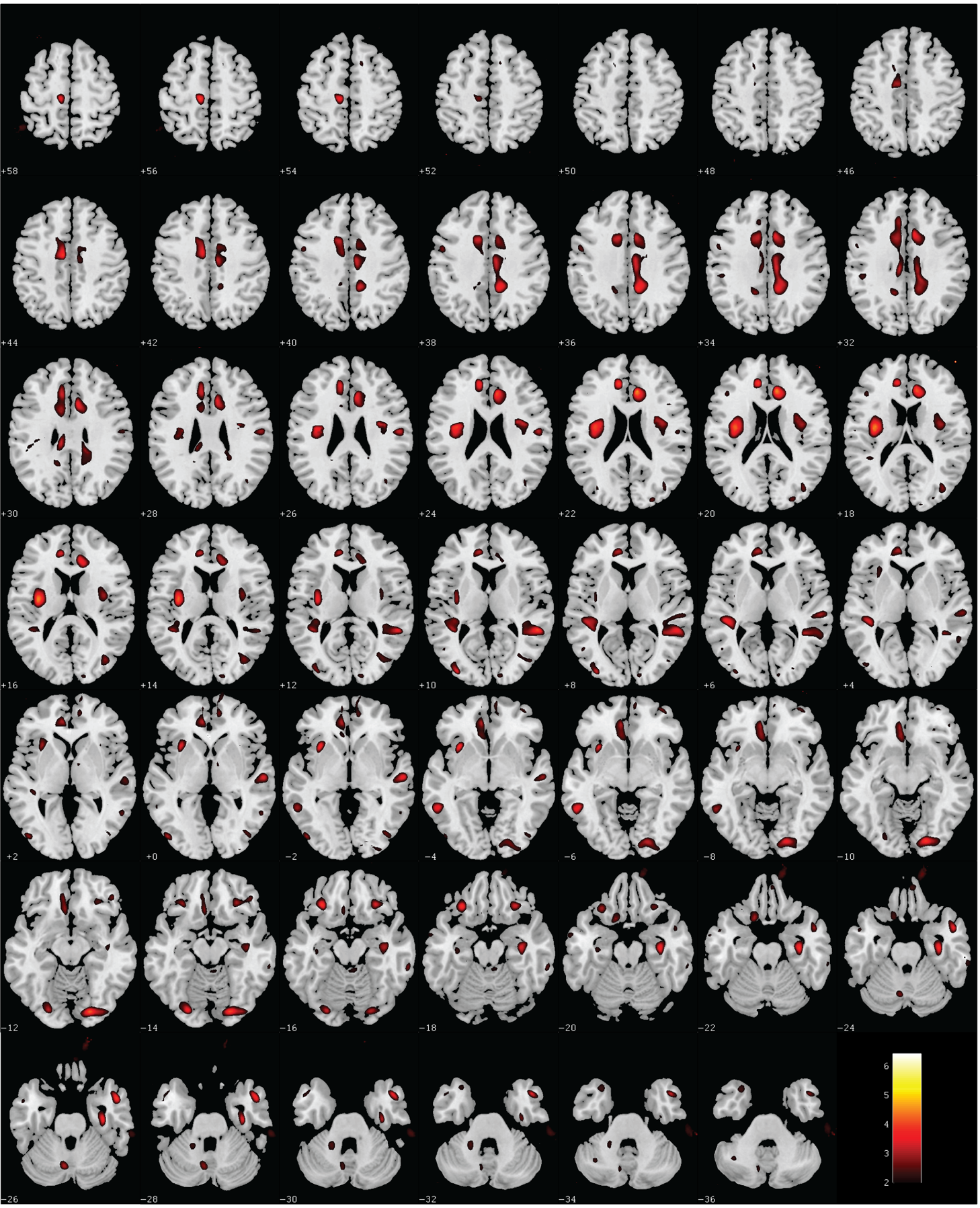

Supplement: Figure S3 — the GMC map associated to deletions at 22q13.31 using a voxel-wise linear regression model. (TIF) [file pone.0052865.s003.tif]
