# Supplementary material for: A Pilot Study on Collective Effects of 22q13.31 Deletions on Gray Matter Concentration in Schizophrenia
Source: PLoS One. 2012 Dec 28;7(12):e52865. doi: 10.1371/journal.pone.0052865 (PMC3532105; doi:10.1371/journal.pone.0052865)
Supplement: Table S1 — 22q13.31 CNVs’ chromosome position (hg18/NCBI36). (DOCX) [file pone.0052865.s004.docx]

Table S1: 22q13.31 CNVs’ chromosome position (hg18/NCBI36)

| Chromosome | starting position | ending position | base pair | copy number | subject type |
| --- | --- | --- | --- | --- | --- |
| 22 | 42,712,496 | 42,713,046 | 551 | 1 | SZ |
| 22 | 42,895,630 | 42,900,679 | 5,050 | 1 | control |
| 22 | 43,514,030 | 43,515,508 | 1,479 | 3 | SZ |
| 22 | 43,514,030 | 43,516,151 | 2,122 | 0 | SZ |
| 22 | 43,514,030 | 43,516,151 | 2,122 | 0 | SZ |
| 22 | 43,514,030 | 43,516,151 | 2,122 | 0 | SZ |
| 22 | 43,514,030 | 43,516,151 | 2,122 | 0 | SZ |
| 22 | 43,514,030 | 43,516,151 | 2,122 | 0 | SZ |
| 22 | 43,514,030 | 43,516,198 | 2,169 | 3 | control |
| 22 | 43,514,442 | 43,515,404 | 963 | 3 | SZ |
| 22 | 43,514,442 | 43,515,920 | 1,479 | 3 | SZ |
| 22 | 43,933,495 | 43,934,937 | 1,443 | 1 | control |
| 22 | 43,933,495 | 43,935,300 | 1,806 | 1 | SZ |
| 22 | 43,933,495 | 43,935,300 | 1,806 | 1 | SZ |
| 22 | 43,933,495 | 43,935,300 | 1,806 | 1 | SZ |
| 22 | 43,933,495 | 43,935,300 | 1,806 | 1 | SZ |
| 22 | 43,933,495 | 43,935,300 | 1,806 | 0 | SZ |
| 22 | 44,032,602 | 44,034,795 | 2,194 | 0 | SZ |
| 22 | 44,032,602 | 44,035,019 | 2,418 | 0 | SZ |
| 22 | 44,032,602 | 44,035,019 | 2,418 | 0 | SZ |
| 22 | 44,032,602 | 44,035,019 | 2,418 | 0 | control |
| 22 | 42,896,308 | 42,896,726 | 418 | 0 | SZ |
