# Supplementary material for: A Pilot Study on Collective Effects of 22q13.31 Deletions on Gray Matter Concentration in Schizophrenia
Source: PLoS One. 2012 Dec 28;7(12):e52865. doi: 10.1371/journal.pone.0052865 (PMC3532105; doi:10.1371/journal.pone.0052865)
Supplement: Text S1 — the CNV detection procedure. (DOCX) [file pone.0052865.s005.docx]

Text S1: the CNV detection procedure

The CNV detection pipeline is illustrated in the following flowchart. For the raw log R ratio (LRR) data, we first perform outlier smoothing, and then apply principal component analysis (PCA) based correction to eliminate variations in the LRR data induced by potential confounding factors. After PCA-correction, samples are excluded if they fail the quality control. The corrected data are then segmented using a circular binary segmentation (CBS) algorithm and a hidden Markov model (HMM) algorithm independently. Those segments reported by both algorithms are flagged as potential CNVs, whose qualities are further investigated through signal-to-noise ratio (SNR). A CNV segment is called only when its SNR passes the preselected threshold. Finally we exclude outlier samples with excessive numbers of CNVs.

Outlier Smoothing

PCA-Correction

Sample Quality Control based on LRR_SD

Circular Binary Segmentation

Hidden Markov Model

Outlier Sample Elimination

Segment Quality Check

*Outlier smoothing:* Outliers manifest as large negative or positive values appearing at only one marker location. An isolated outlier can affect the regional LRR mean, resulting in incorrectly assigned segments. Here we adopt the method introduced by Olshen et al. [[1](#_ENREF_1)] for outlier smoothing. Specifically, it is to replace any local maximum or minimum, which is 4-standard deviation (SD) away from its nearest neighbor in the local 5-marker window, using the value of median ± 2SD.

*PCA-correction:* PCA-correction is employed to remove the variations in the LRR data induced by potential confounding factors (e.g. scanner artifacts or GC-content). Through PCA, LRR data are decomposed into a linear combination of underlying principal components (PCs), with each PC accounting for a certain amount of variance. Pearson correlation or analysis of variance (ANOVA) is then used to assess the associations of PCs with all the potential continuous or categorical confounding factors. Specifically, a PC with a significant association after Bonferroni correction is considered as reflecting a confounding factor. Then the data quality can be improved, theoretically, by removing the effect of this PC.

*Sample quality control:* After the outlier smoothing and PCA-correction, the data quality can still vary dramatically from sample to sample. Therefore, samples whose LRR data exhibit a standard deviation (LRR_SD) greater than 0.28, as recommended in [[2](#_ENREF_2),[3](#_ENREF_3)], are considered as bad samples, and excluded from subsequent analyses.

*Segmentation*: Segmentation is performed with two independent algorithms: CBS [[1](#_ENREF_1)] implemented in MATLAB; and HMM segmentation implemented in PennCNV [[4](#_ENREF_4)]. The default settings are chosen for both algorithms. A segment is flagged as a potential CNV only when it is detected by both algorithms (i.e., the two segments reported by CBS and HMM respectively overlap or are apart by less than 3 markers).

*Segment quality check:* We further evaluate the qualities of potential CNVs based on SNR. For each potential CNV, we extract a number of neighboring markers to cover a comparable length of base pairs, serving as a reference. Then the SNR is calculated as the ratio of LRR mean difference between the potential CNV and the reference over the LRR_SD of the reference. A potential CNV is finally validated if its SNR is greater than 1.4 in case of insertions, or greater than 2 in case of deletions, where the thresholds are estimated empirically based on the LRR means of single insertions and deletions, respectively.

*Outlier Sample Elimination*: Based on the detected CNVs, we eliminate those outlier samples for which an excessive number of CNVs are detected (> 3SD).

1. Olshen AB, Venkatraman ES, Lucito R, Wigler M (2004) Circular binary segmentation for the analysis of array-based DNA copy number data. Biostatistics 5: 557-572.

2. Need AC, Ge D, Weale ME, Maia J, Feng S, et al. (2009) A genome-wide investigation of SNPs and CNVs in schizophrenia. PLoS genetics 5: e1000373.

3. Bucan M, Abrahams BS, Wang K, Glessner JT, Herman EI, et al. (2009) Genome-wide analyses of exonic copy number variants in a family-based study point to novel autism susceptibility genes. PLoS genetics 5: e1000536.

4. Wang K, Li M, Hadley D, Liu R, Glessner J, et al. (2007) PennCNV: an integrated hidden Markov model designed for high-resolution copy number variation detection in whole-genome SNP genotyping data. Genome Res 17: 1665-1674.
